# Breast Lift with and without Implant: A Synopsis and Primer for the Plastic Surgeon

**DOI:** 10.1097/GOX.0000000000003057

**Published:** 2020-10-28

**Authors:** Smita R. Ramanadham, Anna Rose Johnson

**Affiliations:** From the * SR Plastic Surgery P.C., East Brunswick, N.J.; † Division of Plastic and Reconstructive Surgery, Rutgers Robert Wood Johnson Medical School, New Brunswick, N.J.

## Abstract

Breast ptosis is a common occurrence following weight loss, pregnancy, and breastfeeding, or as a consequence of normal aging. This results in loss of a youthful shape and contour of the breast, with a change in the position of the nipple–areolar complex. Mastopexy can restore this youthful appearance and transpose the nipple–areolar complex to a more aesthetic position on the breast. Various techniques exist that address the skin and parenchyma of the breast and are chosen based on the degree of ptosis and skin laxity, as well as the patient’s goals. These techniques all differ in scar burden and risk profile. Additionally, this can be done simultaneously or in a staged manner. In this literature review, we aim to provide an overview of mastopexy procedures, with and without augmentation. Further, we aim to detail recent advancements in technical approaches, and delineate common complications in certain patient demographics. To this end, we performed a literature search with a medical librarian, using PubMed/Medline to identify pertinent literature. In the context of the review, we discuss important considerations in patient selection and counseling to set expectations and ultimately, optimize surgical outcome and patient satisfaction.

Breast ptosis, sagging, or descent of the breast is a common occurrence with age or following pregnancy, breastfeeding, or weight loss. Ptosis can be classified using the Regnault classification system. This commonly used system describes ptosis by the relative position of the nipple–areolar complex (NAC) and the inframammary fold (Table 1).^1–5^ As the breast enlarges with age, weight, or hormonal changes, the skin envelope, supporting ligaments, and ducts stretch. These then fail to retract when the volume decreases. As a result, breast position is lower on the chest wall, breast contour changes, and upper pole fullness is decreased.^1^ Mastopexy seeks to restore a youthful shape and contour of the breast by transposing the NAC to a more desirable position on the breast mound. This can be accomplished via various approaches that differ with respect to skin incisions, parenchymal redistribution and fixation, auto-augmentation, or augmentation with implant.^1,4^ Augmentation mastopexy has further shown reliable results when performed in a staged manner.^6^

**Table 1.** Regnault’s Classification of Breast Ptosis Based on the Position of the NAC Relative to the Inframammary Fold

Scale	Criteria
Pseudoptosis	NAC is above the IMF
Type I (mild)	NAC is at or 1 cm below the IMF
Type II (moderate)	NAC is 1–3 cm below the IMF
Type III (severe)	NAC is at the lowest portion of the breast

NAC, nipple–areola complex; IMF, inframammary fold.

Reprinted with permission from Wolters Kluwer Health from *Plast Reconstr Surg.* 2011;127(4):91e–102e.

## Methodology

A comprehensive literature search using PubMed/MEDLINE during the time period of 1980–2020 was performed with a medical librarian to identify pertinent literature, which described common and emerging techniques for mastopexy, with and without implant. The search terms included “breast lift,” “breast lift with implant,” “augmentation mastopexy,” and “mastopexy.” We aimed to provide an overview of literature and focus on key clinical factors for physicians to guide patient selection and preoperative counseling, and to review outcome metrics. Articles that described a mastopexy technique in human subjects were identified and considered eligible for inclusion.

The American Society of Plastic Surgeons has reported that breast lifts or mastopexy has increased by 70% since 2000.^7^ As this procedure continues to grow in popularity, it is important to better define the patient population that may benefit most from available approaches.

### Patient Education

The pre-operative consultation allows the surgeon to understand key components in determining the best surgical approach. It is important to inquire about patient-specific concerns about breast size and shape to facilitate healthy patient–provider dialogue, reduce any potential for misinformation, and promote discussion of realistic surgical outcomes.

Patient goals regarding breast size and upper pole fullness will first need to be discussed. Importantly, eliciting information regarding patient concerns about breast shape or size can further determine the best course of treatment.^8^ If a patient is happy with the volume and upper pole fullness, a mastopexy alone may suffice. It is important to counsel the patient that a decrease of 1 cup size is expected with a mastopexy alone.^9^ However, if a patient instead desires restoration of upper pole volume or an increase in breast size, a mastopexy should be combined with an augmentation in a 1- or 2-stage manner, depending on the amount of nipple elevation and vertical excess. While performed less often than breast augmentation and breast reduction, the litigation rate remains relatively high.^1^ This underscores the importance of individualizing patient counseling regarding associated risks and expected outcomes.

### Patient Selection

A comprehensive medical and surgery history should be obtained. A focused breast history should include history of and/or desire for breastfeeding, personal or family history of breast cancer, prior surgeries, oral contraceptive use, and any bleeding diathesis. Smoking history is an important component of the selection process because it confers a greater risk of complications. Existing guidelines do not support operating on current smokers.^10^ Moreover, specific considerations in the massive weight loss patient include any prior surgeries that may be associated with nutritional deficiencies. These should be addressed before surgical intervention.^11^ Factors including prior breast surgeries should be obtained to avoid complications associated with compromised blood supply to the NAC and surrounding skin envelope.^8^

### Physical Examination

**Fig. 1.**
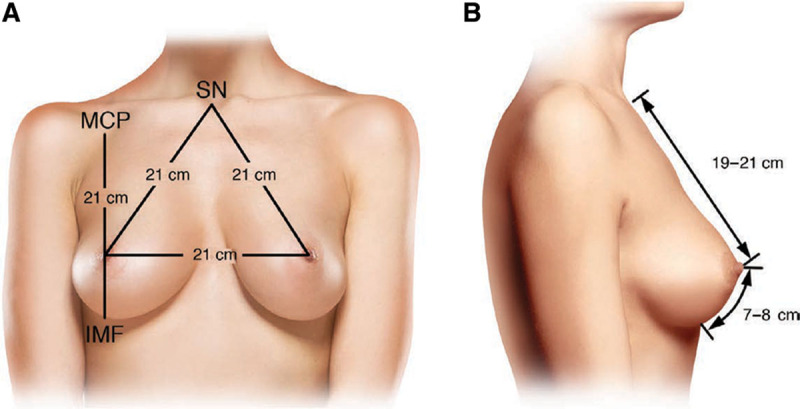
Standard preoperative breast markings. Frontal (A) and lateral (B) views of standard breast measurements. Reprinted with permission from Wolters Kluwer Health from *Plast Reconstr Surg*. 2018;142(5):742e.

The physical examination should include assessment of the breast position on the chest, degree of ptosis, skin and parenchymal quality, areolar size, and evaluation of any asymmetries. Bilateral measurements should be documented. The approach to obtaining these measurements in a standardized fashion has already been described (Fig. 1).^1,2,4,12^ A general preoperative cardiac and respiratory examination needs to be performed to assess the overall safety of the procedure and anesthesia. In most cases, primary care clearance and preoperative blood work should be obtained.

## Description of Procedure

Mastopexy techniques can be discussed in relation to the skin incision, parenchymal redistribution, and use of an implant. The ideal technique is chosen based on the patient’s examination and goals of surgery. In this review, we will focus on the most commonly used approaches and detail any new advancements or considerations.

### Surgical Techniques

#### Crescent Mastopexy

**Fig. 2.**
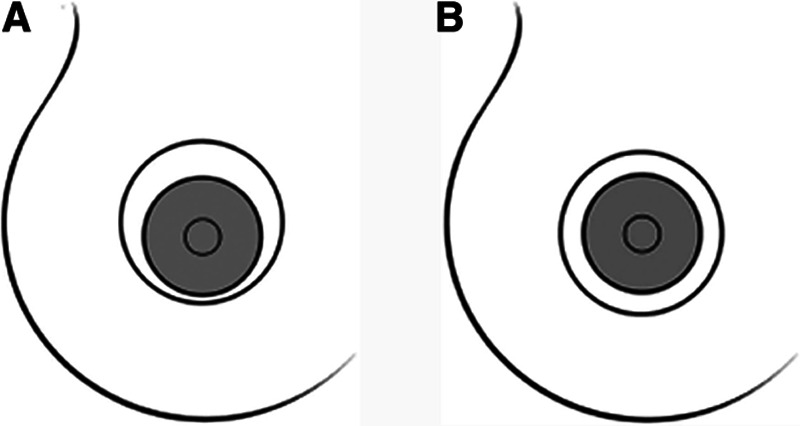
Illustration of two different mastopexy patterns. A, Eccentric mastopexy pattern. B, Periareolar (donut) mastopexy pattern. Reprinted with permission from Wolters Kluwer Health from *Plast Reconstr Surg.* 2011;127(4):91e–102e.

The crescent mastopexy (Fig. 2)^13^ is an eccentric circumareolar mastopexy without areolar mobilization or purse string. It can address minor asymmetries and has been used to minimize scars for patients with small to moderate ptosis.^14,15^ However, risks include poor scarring and oval deformity of the NAC. Efforts to modify the technique and minimize complications by excising parenchyma on either side of the areola and/or combination with an implant have been attempted, with varying success.^14^

#### Circumareolar

The circumareolar or “donut” mastopexy (Fig. 2)^13^ may be a good option if the NAC is not wide and does not need to be raised greater than 2 cm. This procedure was intended to minimize scarring of periareolar area, preserve NAC sensation, and shorten the duration of surgery.^16^ Risks are minimized if the outer diameter is <7 cm and the inner is typically placed within the areola or just outside. The ratio of the outer diameter to inner should not exceed 2:1. Risks include pleating, flattening, wide scaring, and areolar spreading. This can be decreased by using a permanent purse string suture.^4^ Further, this can be combined with an implant in a single-stage to raise the NAC and increase breast volume.^17^

The Benelli procedure allows for parenchymal repositioning and is a true circumareolar mastopexy. This technique is based off of a superior pedicle. The skin is undermined to expose the underlying breast tissue, the parenchyma is incised, and medial and lateral flaps are mobilized and crossed in the midline. Non-absorbable sutures around the areolar incision are used.^18^ This results in narrowing and coning of the breast.^4,13,19^

#### Vertical

**Fig. 3.**
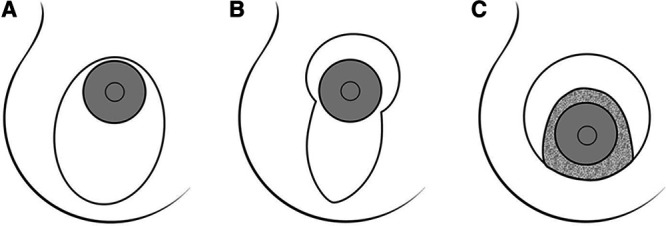
Diagram illustrating the approaches to correct breast ptosis: A, vertical scar technique; B, Lejour technique; C, Hammond technique. Reprinted with permission from Wolters Kluwer Health from *Plast Reconstr Surg*. 2004;114:1622–1630.

A more powerful technique with an increased ability to resect skin and raise the nipple is through a vertical mastopexy (Fig. 3).^1–4,13^ This results in less circumareolar skin tension, as the incisions are closed around the NAC and inferiorly to the inframammary fold (IMF).^1,4^ It has been described as a good option for grade I–III ptosis. Increased splay angle of the vertical limbs and limb length result in a greater nipple movement and coning of the breast.^20,21^

The Lassus procedure is a vertical mastopexy without undermining. This technique was intended to produce long-lasting results with minimal scarring in patients with hypertrophic, ptotic breasts. The inferior skin, fat, and parenchyma are resected en bloc while transposing the nipple superiorly. Pillars are then closed inferiorly.^22^ Important principles must be respected to maximize success, including transposition of the nipple no more than 9 cm.^22^ If this cannot be accomplished, use of a laterally based flap should be considered.

The short scar periareolar inferior pedicle mammaplasty (SPAIR), developed by Hammond, is a technique in which the skin is resected in a vertical manner while transposition of the NAC occurs based on inferior pedicle.^23^ This technique has different options for periareolar closure, including the use of a Gore-Tex suture and pin-wheel or an interlocking pattern of periareolar suture placement.^23^ This approach is not traditionally combined with implant placement.

Medial pedicle vertical mammaplasty (Hall-Findlay) is a procedure in which the NAC is based on a medial pedicle, while lateral and medial tissue is excised or positioned superiorly. Pillars are closed and the skin is closed in a vertical manner.^24^ This technique can be used in patients with all grades of ptosis. It confers the advantage of ptosis correction and removal of glandular tissue while augmenting structural support with pillar unification and closure. However, final results are often delayed, and persistent asymmetries have been described.^8^

#### Y-scar

**Fig. 4.**
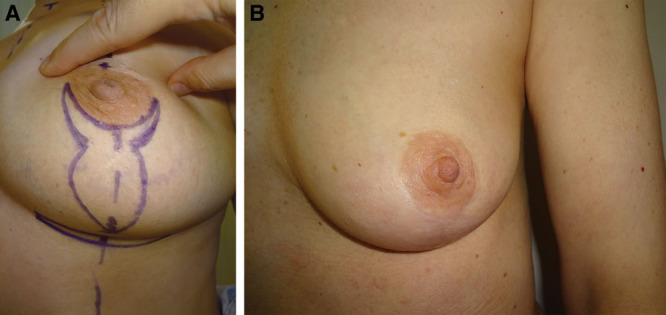
Photograph displaying the fish mastopexy technique. A, Preoperative markings. B, Postoperative outcome. Reprinted with permission from Wolters Kluwer Health from *Plast Reconstr Surg.* 2012;129(5):865e–866e.

The Y-Scar vertical mastopexy eliminates the need for a superior circumareaolar incision but otherwise is similar in approach to a conventional vertical mastopexy.^4^ Its modern role and utility is limited because it requires that the patient has normal NAC diameter and ideal nipple position. However, in the patient who meets these specifications, this technique offers reproducible outcomes, with the advantage of minimal scar burden.^4^ This technique results in tightening of the lower pole and requires minimal nipple adjustment. This may also be an appropriate technique for the patient who desires simultaneous mastopexy with augmentation.^25,26^ Since its inception, it has modified and broadened in its application to women with skin laxity and pseudoglandular ptosis. The “fish mastopexy” should be considered for women with an NAC at the inframammary fold and ptosis of glandular breast tissue below the IMF (Fig. 4).^26^

#### Inverted-T

**Fig. 5.**
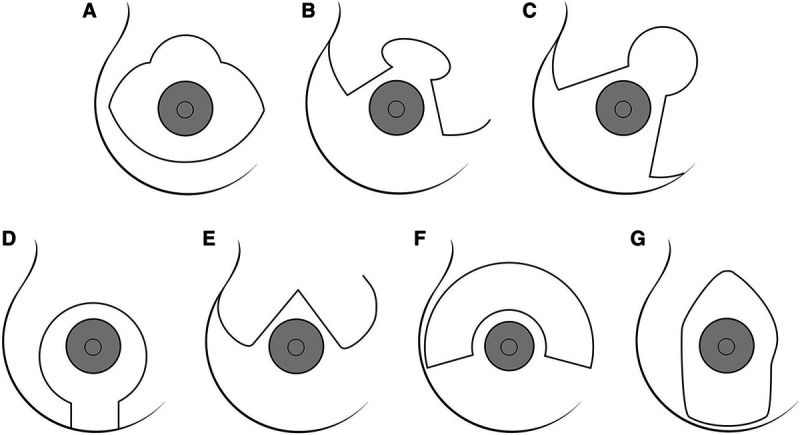
Illustration of the inverted-T scar technique: A, Strombeck; B, Flowers; C, Nicolle; D, Peixoto; E, Pitanguy; F, Wise; G, Marchac. Reprinted with permission from Wolters Kluwer Health from *Plast Reconstr Surg*. 2004;114:1622–1630.

The inverted-T technique (Fig. 5)^19^ is a flexible, adaptive technique that allows for resecting excess skin and elevating the nipple.^27^ The technique is ideal for grade III or severe ptosis or those with poor skin quality. Commonly, a wise pattern skin excision is used; however, other patterns are shown in Figure 3.^1,4^ This technique has been associated with success in the patient with massive weight loss as the lateral horizontal limbs can be designed to extend as far as the axilla.^4^ It should be noted that an important caveat for this procedure is its association with significant scar burden with horizontal, vertical, and areolar components.^10^ Further, the complication of skin necrosis at the inverted-T junction should be noted.^28^

#### Adjunct Techniques: Parenchymal Redistribution and Mesh

To optimize the duration of operative results, parenchymal re-distribution techniques, fixation, use of mesh, and auto augmentation have been incorporated into mastopexy procedures. No study to date demonstrates superiority of one technique over the other.^1,4^ Rearrangement of the parenchyma has been to reinforce the lower pole and increase breast projection. For example, in superior pedicle-based approaches, the inferior parenchyma can be tunneled under a pectoral sling, folded under the pedicle and sutured to the pectoralis fascia, or folded over to create a sling for support of the lower pole. Auto augmentation techniques use lateral or medial breast tissue to create a projected breast mound by rotation of the tissue.^29–31^

Adjunctive materials (including acellular dermal matrix and mesh) have been used in mastopexy procedures.^32^ In one study by Adams et al., resorbable monofilament mesh was used to reinforce the lower pole of the breast. At one year of follow up, the authors reported minimal stretch of the lower pole, suggesting that this adjunct may confer utility in patients with larger breasts and skin laxity.^33^ It should be noted that despite modifications in technique or use of adjuncts, recurrent ptosis and bottoming out are commonly observed postoperatively. Parenchymal and skin laxity are the underlying causes of these that cannot be addressed surgically.^33^ Research efforts should focus on evaluation of emerging post-operative therapies (ie, non-invasive skin tightening modalities),^10^ which may be successful in tempering this result.

#### Augmentation Mastopexy

**Fig. 6.**
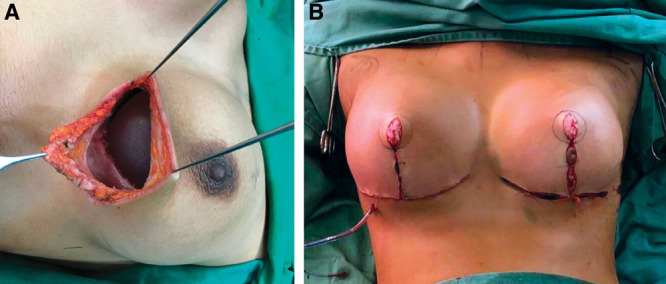
Photograph showing lift and augmentation at a single time approach. A, First step. B, Last step. Reprinted with permission from Wolters Kluwer Health from *Plast Reconstr Surg–Global Open.* 2019;7(11):e2523.

Thus far, we have described common mastopexy approaches and specified when they may be combined with augmentation in either a single or staged result. Overall, augmentation mastopexy is a good option for patients that lack volume and desire increased upper pole fullness or increased overall breast size. The addition of an implant is challenging because it creates 2 opposing forces. Augmentation increases volume and the skin envelope, while mastopexy repositions the nipple and removes excess skin. Together, tension on the wound increases, as well as, gravitational forces on the breast. This can significantly compromise the NAC, especially with greater nipple movement and larger implants. Emerging single-step techniques include the Lift and Augmentation at Single Time technique^34^ for augmentation-mastopexy. This approach utilizes a standardized 4-step sequence that augments inferolateral implant support to prevent lower pole ptosis (Fig. 6).

#### Massive Weight Loss Patient

The patient presenting after massive weight loss poses a unique challenge to the plastic and reconstructive surgeon. These patients^1,35,36^ have physical presentations, including poor skin elasticity and lack of a discrete IMF and psychological considerations, which require a comprehensive treatment approach.^35,37^ Patients usually present with severe ptosis, asymmetry, loss of upper pole volume, medialization of the NAC, lateral chest wall skin excess or lipodystrophy, and a loose IMF. Recurrent ptosis and poor scarring remain common procedural outcomes. If a patient has enough volume, auto-augmentation or dermal suspension techniques can be used. If volume is insufficient, implants should be added. In a study by Coombs et al, 30 patients with grade III ptosis were evaluated between 2003 and 2011.^35^ Five patients (16.7%) developed ptosis within 3 months, and implant malposition was observed in 61.9% of patients. Suggested techniques to reduce this include an inverted-T resection, stabilization of the IMF with suturing to the periosteum, use of textured implants or implants with tabs for fixation, or use of acellular dermis.^35^

## AVOIDING AND MANAGING MOST-DANGEROUS COMPLICATIONS

Complications^1,4^ can be tissue-related or implant-related and depend on whether a mastopexy is performed alone, in combination with an augmentation, or as a single- or a dual-staged approach. As with all procedures, the key to decreasing these rates is appropriate patient selection and counseling regarding individualized risks, expectations, and outcomes. Risk factors associated with an increased complication profile (including obesity and smoking) should be discussed with the patient. If possible, optimization of comorbidities and cessation of smoking should occur before surgical intervention.^38^

Complications and rates can vary based on technique and can help guide selection of the optimal approach for the patient. The most common complications described are associated with circumareolar and inverted-T mastopexy techniques. In circumareolar mastopexies, areolar herniation, purse-string breakage, knot exposure, and a palpable ring are commonly observed.^10,39^ To address this, deep dermal sutures are recommended to avoid some of the complications related to the purse-string suture. Other complications include widened scars and NAC, central flattening, and pleating.^8^ Techniques to address this include conversion to a vertical mastopexy or, in the case of augmentation mastopexies, downsizing of the implant. Circumareolar mastopexies result in an overall complication rate of 41.5% compared with 9.7% in vertical mastopexies and 14% with an inverted-T resection.^4,40^ Circumareolar techniques also tend to have higher revision rates, while vertical have increased asymmetries, and inverted-T are seen to more commonly bottom out.

The addition of an implant further adds to the list of risks. A study by Doshier et al evaluated complication rates comparing augmentation mastopexy, augmentation alone, and mastopexy alone. They found that tissue-related complications were more common in combined procedures compared with mastopexy alone. While the mastopexy group did not provide enough data for quantification, the revision rate was 7.97% and 12.4% in individual and combination procedures, respectively.^39^ These authors describe that key considerations in optimizing success of a combined approach include careful attention to the original areola size and its position with respect to the chosen pattern for mastopexy (ie vertical or Wise) and preoperative identification and marking of the new NAC position (for subsequent implant placement and manipulation).^39^ The authors argue that the reoperation rate of 12.4% is significantly less than that for a single-staged procedure (100%). However, existing literature supports the careful selection of patients who would benefit from this approach due to the complication profile. In their meta-analysis, Khavanin et al. reported a total complication rate of 13.1% in augmentation mastopexy, with recurrent ptosis being the most common complication (5.2%). Other complications included unappealing scarring (3.7%), capsular contracture (3%), and asymmetry (2.9%).^41^ Further, findings from this study mirror those of Doshier et al, as tissue-related complications were more common than implant-related complications. The authors contend that weight of the implant contributed especially to recurrent ptosis.^41^

**Fig. 7.**
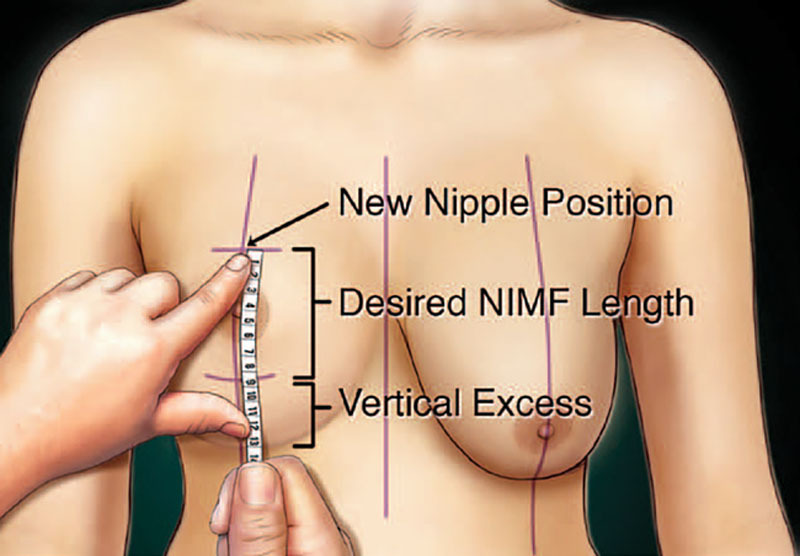
Illustration showing the evaluation of vertical excess. Vertical excess is determined by marking the desired nipple position and then measuring under stretch the desired nipple-to-inframammary fold (*NIMF*) length based on breast width. The remaining skin to the existing inframammary fold is the vertical excess. Reprinted with permission from Wolters Kluwer Health *Plast Reconstr Surg*. 2014; 134:215.

**Fig. 8.**
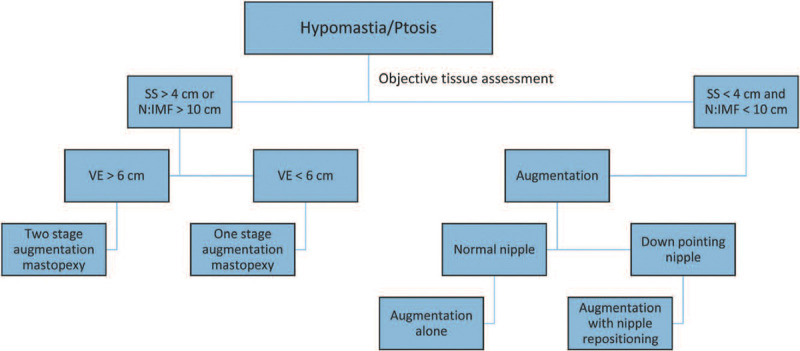
Tissue-based triad algorithm for guiding surgical planning. SS, skin stretch; N:IMF, nipple-to-inframammary fold distance; VE, vertical excess. Reprinted with permission from Wolters Kluwer Health from *Plast Reconstr Surg*. 2014; 134:215.

To prevent these complications, the use of small implants is recommended with moderate elevation of the nipple and a broad pedicle in augmentation mastopexy.^42^ Conversely, a tissue-based triad approach is suggested for choosing appropriate patients for combined procedures. A single stage is recommended for vertical excess of <6 cm and a staged procedure is recommended for >6 cm in those patients with skin stretch of >4 cm and N-IMF > 10 cm.^12^ Vertical excess is defined as the vertical distance from the surgically desired IMF based on the base width, ideal nipple position, and the preoperative fold (Figs. 7, 8). While risks are significant, some argue that a revision rate of 10%–30% is ultimately always less than a reoperation rate of 100% for the second stage. This results in less cost to the patient and in less risk of anesthesia.

## PEARLS AND PITFALLS

Patient selection is paramount to meet patient goals, optimize surgical outcomes, and decrease complications. Patients who have risk factors increasing their complication rate require early counseling and discussion of a heightened risk of complications. Techniques should be chosen based on patient anatomy of nipple position, skin excess, and breast parenchyma. Informed consent should include operative risks and complications, including bleeding, hematoma, infection, delayed wound healing or dehiscence, loss of nipple sensation, inability to breast feed postoperatively, scarring, asymmetry, and recurrent ptosis. Discussions should be individualized to each patient. For example, in the patient with massive weight loss, discussion of reoperation rate should be emphasized. Further, in a patient requiring vertical components, communication of expected scarring and scar patterns is appropriate.^4^

Photographs should be obtained to document preoperative status and can be used for further discussions with the patient during their operative course. More importantly, if the patient’s goals and expectations do not align with what the surgery can realistically accomplish, surgery should be avoided.

## WHAT PATIENTS SHOULD KNOW BEFORE HAVING THIS PROCEDURE

It is important for patients to have a comprehensive preoperative understanding of this procedure and expected outcomes (Table 2). They should understand that mastopexy with and without augmentation can improve the shape and appearance of the breast that can restore a youthful contour and place the NAC in a more ideal location. Upper pole fullness can also be achieved with the combined use of an implant. Surgery cannot, however, change the quality of the skin or tissue. This, oftentimes, results in recurrent ptosis and is especially true in those instances when an implant is added, as the weight of the implant itself contributes to this ptosis. Furthermore, there is a tradeoff between shape and scar. With increased degrees of ptosis and skin excess, there will be a larger scar burden. Despite this, mastopexy can successfully address the consequences of aging, breastfeeding, and weight loss in the right candidate.

**Table 2.** Surgical Approaches and Results^4,5,8,10,14^

Approach	Indicated Population	Conducive to Combined Mastopexy Augmentation?	Goals	Complications (immediate/delayed)	Long-term Expectations
Periareolar “Crescent Mastopexy”	Grade I/II ptosis	Yes	Address minor NAC asymmetries	Areolar herniation Purse-string breakage Palpable ring	Flattening and deprojection of the breast over time; scar hypertrophy
SPAIR	Grade I–III ptosis	Yes*	Ptosis correction; removal of glandular tissue (SPAIR), restoration of projection	Periareolar widening, pleating	Bottoming out; delayed and persistent asymmetries
Hall-Findlay	I–III ptosis	Yes	Ptosis correction, improved breast projection	Scarring	Final appearance takes time
Inverted-T	Grade III ptosis	Yes	Breast mound and lower pole elevation to restore youthful contour	Skin necrosis at the T-junction; hypertrophic scarring	Bottoming out over time
Y-Scar	Normal NAC diameter and ideal nipple position. Minimal to no ptosis	Yes	Improved projection with minimal scarring	Inferior areolar fullness; seroma formation	Bottoming out over time

*Yes, although not traditionally performed.^8^

## LIMITATIONS

This article was designed as a literature review that identifies common and emerging mastopexy procedures, with and without implant. This was not a systematic literature review but a synopsis of key and emerging techniques. We aimed to provide the clinician with a pertinent overview and guide for patient selection, education, and outcomes analysis.
